# Nonlinear Dynamics of the Introduction of a New SARS-CoV-2 Variant with Different Infectiousness

**DOI:** 10.3390/math9131564

**Published:** 2021-07-03

**Authors:** Gilberto Gonzalez-Parra, Abraham J. Arenas

**Affiliations:** 1 Department of Mathematics, New Mexico Tech, Socorro, NM 87801, USA; 2 Departamento de Matemáticas y Estadística, Universidad de Córdoba, Montería 230002, Colombia

**Keywords:** SARS-CoV-2 virus, global stability analysis, Lyapunov functions, variants, basic reproduction number

## Abstract

Several variants of the SARS-CoV-2 virus have been detected during the COVID-19 pandemic. Some of these new variants have been of health public concern due to their higher infectiousness. We propose a theoretical mathematical model based on differential equations to study the effect of introducing a new, more transmissible SARS-CoV-2 variant in a population. The mathematical model is formulated in such a way that it takes into account the higher transmission rate of the new SARS-CoV-2 strain and the subpopulation of asymptomatic carriers. We find the basic reproduction number ${𝓡}_{0}$ using the method of the next generation matrix. This threshold parameter is crucial since it indicates what parameters play an important role in the outcome of the COVID-19 pandemic. We study the local stability of the infection-free and endemic equilibrium states, which are potential outcomes of a pandemic. Moreover, by using a suitable Lyapunov functional and the LaSalle invariant principle, it is proved that if the basic reproduction number is less than unity, the infection-free equilibrium is globally asymptotically stable. Our study shows that the new more transmissible SARS-CoV-2 variant will prevail and the prevalence of the preexistent variant would decrease and eventually disappear. We perform numerical simulations to support the analytic results and to show some effects of a new more transmissible SARS-CoV-2 variant in a population.

## 1. Introduction

The world is suffering one of the worst pandemics in history. The spread of the SARS-CoV-2 virus started at the end of the year 2019 and has now affected the whole world from a variety of points of view. The COVID-19 pandemic has caused more than 160 million confirmed cases and more than 3.2 million deaths (May 2021) [1,2]. It is important to remark that the number of cases is underestimated due to lack of tests and asymptomatic cases among other reasons [3–11]. Several variants of the SARS-CoV-2 virus have been discovered and some of these new SARS-CoV-2 variants have been of concern due to their higher transmissibility [12–18]. These new more transmissible SARS-CoV-2 variants can have a great impact on the number of infected cases, prevalence, hospitalizations and deaths. The people, researchers, and media are concerned about what the consequences will be of having a more transmissible SARS-CoV-2 variant [12,18–21]. It has been observed that not all the countries have been able to start a strong vaccination program and therefore these countries are facing the introduction of SARS-CoV-2 variants that are more transmissible.

Mutations of viruses are common and, as a consequence, SARS-CoV-2 can acquire mutations that allow the virus to spread better and provide immunological resistance [12–17,22]. In 2020, a new variant of the SARS-CoV-2 virus was discovered in England, and has been named the VOC-202012/01 of lineage B.1.1.7. [23–25]. This new SARS-CoV-2 variant increased the number of infected cases and deaths in England. Test results showed that the new SARS-CoV-2 variant, VOC-202012/01, was prevalent and its proportion increased at the end of 2020 in England [26]. Several studies have found that the new SARS-CoV-2 variant VOC-202012/01 is more transmissible than the previously prevalent variants [24,27–29].

Based on the appearance of new SARS-CoV-2, it is important to construct mathematical models to study the potential consequences of these new variants. Mathematical models have been widely used to investigate and understand the dynamics of many infectious diseases [30–39]. Mathematical models provide useful insight into the nonlinear complex phenomena [31,40,41]. Thus, mathematical models are helpful for gaining knowledge and providing scientific support to decide which are the most suitable public health policies. A variety of mathematical models have been developed to study and aid in the control of the spread of the SARS-CoV-2 virus in the population and predicting hospitalizations [42–55]. The proposed models vary in different ways, including assumptions, methodology, techniques and approaches [50,56]. However, our proposed deterministic model is different from others since we consider two different SARS-CoV-2 variants, as well as asymptomatic cases for both variants.

In this study, we construct a compartmental mathematical model based on differential equations to study the effect of introducing a new more transmissible SARS-CoV-2 strain into a population. Agent-based models might be more suitable, but have the difficulty of a greater number of parameters and uncertainties. Another advantage of the differential equation model is that it allows for mathematical analysis over the long term, which provides useful insight into the short term, as well. The differential equation based model we propose belongs to the general class of positive polynomial systems that are applied in many fields [57,58]. The mathematical model is formulated in such a way that it takes into account the higher transmission rate of the new SARS-CoV-2 variant and the asymptomatic individuals.

The paper is organized as follows: in Section 2, we present the mathematical model of SARS-CoV-2 transmission and disease progression and some preliminary results about the positivity of the solutions. Section 3 is devoted to stability mathematical analysis, including local and global stability analysis. In Section 4, the numerical simulation results using the constructed mathematical model of SARS-CoV-2 transmission are shown, and the last section is devoted to the conclusions.

## 2. Mathematical Model of SARS-CoV-2 Spread

We constructed a compartmental model based on a deterministic system of nonlinear differential equations that considers two variants of SARS-CoV-2. This situation has been common in several countries, where there is a prevalent preexistent SARS-CoV-2 variant and then a second variant such as the VOC-202012/01 of lineage B.1.1.7 is introduced in the population.

**Figure 1.**
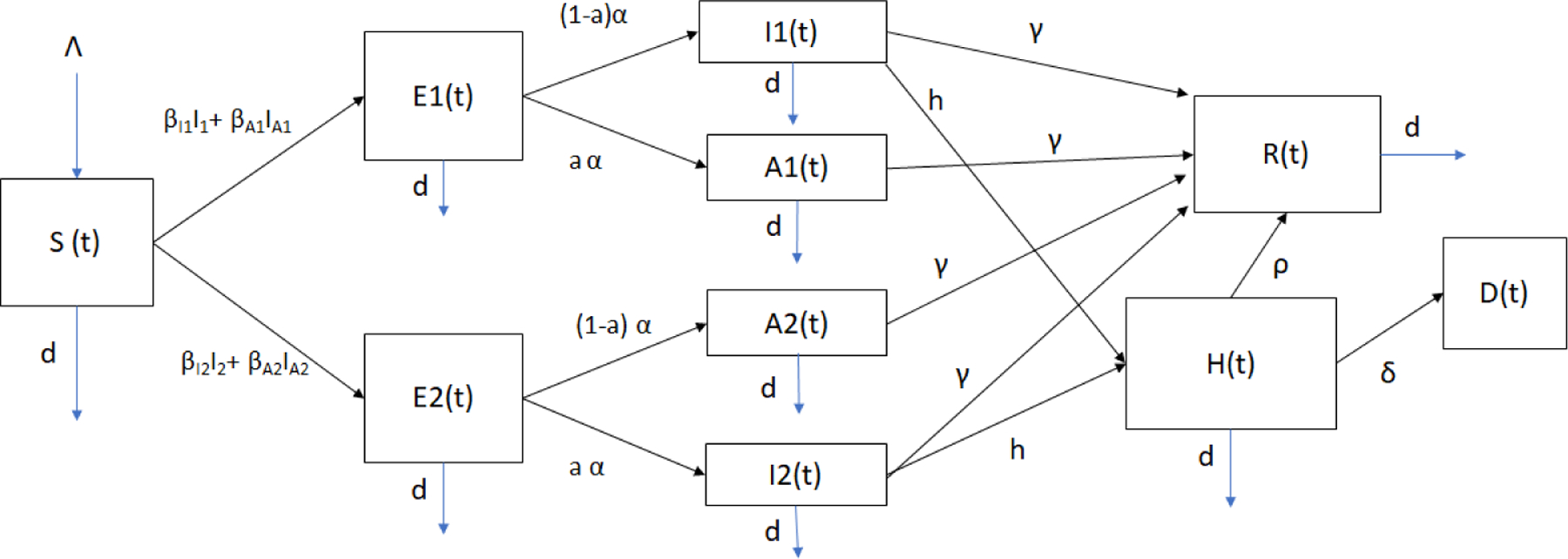
Diagram of the COVID-19 mathematical model (1). This shows the transition of individuals between epidemiological classes. *S*(*t*) is the susceptible class, *E*_1,2_(*t*) are the two classes of latent individuals for the two SARS-CoV-2 variants, *I*_1,2_(*t*) represents two classes of infectious individuals, *A*_1,2_(*t*) are the asymptomatic individuals (one for each variant), *H*(*t*) represents the hospitalized individuals, *R*(*t*) represents the recovered and *D*(*t*) is the number of deaths.

The model includes individuals in the susceptible (*S*(*t*)), latent (*E*(*t*)), infected (*I*(*t*)), asymptomatic (*A*(*t*)), and hospitalized (*H*(*t*)) classes, as shown in Figure 1. The transition of individuals from one class to another depends on the stage of the disease. The mathematical model also assumes that individuals can only get one SARS-CoV-2 variant and there is no co-infection. The individuals can be in two disjoint groups related to disease progression: infected with variant one and infected with variant two. The model also assumes that individuals infected with one SARS-CoV-2 variant have full immunity against the other variant due to the adaptive immune response [59–62]. The model has a constant recruiting rate (births) Λ to the susceptible (*S*(*t*)) class. The transmission rate from infected individuals with variant *i* (*I*_*i*_(*t*)) to susceptible individuals (*S*(*t*)) is given by $\beta_{A_{i}}$. The transmission rate from asymptomatic individuals with variant *i* (*A*_*i*_(*t*)) to susceptible individuals (*S*(*t*)) is given by $\beta_{I_{i}}$ The model includes individuals in the latent stage (either with variant one or variant two) who are not yet infectious. The individuals remain in the latent phase (*E*(*t*)) for a certain time with mean *α*. The individuals in classes *E*_1_(*t*) and *E*_2_(*t*) then transit into the infective symptomatic (*I*_1_(*t*) or *I*_2_(*t*)) or asymptomatic classes (*A*_1_(*t*) or *A*_2_(*t*))), where they are able to transmit the SARS-CoV-2 to other individuals. The infected people stay in the infectious phase for a certain time with mean *γ*. The asymptomatic individuals transit to the recovered class at a rate of *γ*. However, infected individuals with symptoms might also transit to the hospitalized class (*H*(*t*)), as can be seen in Figure 1.

In the mathematical model it is assumed that hospitalized individuals can die due the COVID-19 disease [50,63–66]. Some scientific literature has mentioned that the antibody titers decline over time in individuals who have recovered from COVID-19, particularly in those who were asymptomatic [67]. In the constructed mathematical model, we do not consider that recovered individuals can get reinfected by going back to the susceptible class. This assumption seems plausible for a relatively short period of one year.

The proposed mathematical model has parameters (depicted beside the arrows in Figure 1) related to the severity of the disease, which include the mortality rate. The mathematical model is formulated as follows:

(1) $$\dot{S}(t)=\text{Λ}-dS(t)-\left(\beta_{I_{1}}I_{1}(t)+\beta_{A_{1}}A_{1}(t)+\beta_{I_{2}}I_{2}(t)+\beta_{A_{2}}A_{2}(t)\right)S(t),\;{\dot{E}}_{1}(t)=\left(\beta_{I_{1}}I_{1}(t)+\beta_{A_{1}}A_{1}(t)\right)S(t)-(d+\alpha )E_{1}(t),\;{\dot{I}}_{1}(t)=(1-a)\alpha E_{1}(t)-(d+h+\gamma )I_{1}(t),\;{\dot{A}}_{1}(t)=a\alpha E_{1}(t)-(d+\gamma )A_{1}(t),\;\dot{H}(t)=hI_{1}(t)+hI_{2}(t)-(d+\delta +\rho )H(t),\;\dot{R}(t)=\gamma \left(I_{1}(t)+I_{2}(t)+A_{1}(t)+A_{2}(t)\right)+\rho H(t)-dR(t),\;{\dot{E}}_{2}(t)=\left(\beta_{I_{2}}I_{2}(t)+\beta_{A_{2}}A_{2}(t)\right)S(t)-(d+\alpha )E_{2}(t),\;{\dot{I}}_{2}(t)=(1-a)\alpha E_{2}(t)-(d+h+\gamma )I_{2}(t)\;{\dot{A}}_{2}(t)=a\alpha E_{2}(t)-(d+\gamma )A_{2}(t),\;\dot{D}(t)=\delta H(t),$$

where *N*(*t*) = *S*(*t*) + *E*_1_(*t*) + *I*_1_(*t*) + *A*_1_(*t*) + *H*(*t*) + *R*(*t*) + *E*_2_(*t*) + *I*_2_(*t*) + *A*_2_(*t*) and the initial conditions

(2) $$S(0)>0,E_{1}(0)\geq 0,I_{1}(0)\geq 0,A_{1}(0)\geq 0,H(0)\geq 0,R(0)\geq 0,\;E_{2}(0)\geq 0,I_{2}(0)\geq 0,A_{2}(0)\geq 0,D(0)\geq 0.$$

This model contains ten variables that represent susceptible individuals (*S*(*t*)), two classes of latent individuals (*E*_1,2_(*t*)), two classes of infectious individuals (*I*_1,2_(*t*)), two classes of asymptomatic individuals (*A*_1,2_(*t*)), hospitalized *H*(*t*), recovered *R*(*t*) and deaths *D*(*t*). Individuals in classes *E*_*i*_(*t*), *H*(*t*), *R*(*t*) and *D*(*t*) do not transmit the infection. The parameters inherent to the model (1) are shown in Table 1, and the transition of individuals between subpopulations is shown in Figure 1.

### Positivity and Boundedness of Solutions

All the variables of the mathematical model (1) represent the number of individuals. Therefore, we need to guarantee that the solutions exist, and are positive and bounded. By the fundamental theory of differential equations [74,75], we can check that the solution of the system (1) with the initial condition exists for all *t* ≥ 0 and it is unique. Since variable *D* is decoupled, without loss of generality, we set the following system:

(3) $$\dot{S}(t)=\text{Λ}-dS(t)-\left(\beta_{I_{1}}I_{1}(t)+\beta_{A_{1}}A_{1}(t)+\beta_{I_{2}}I_{2}(t)+\beta_{A_{2}}A_{2}(t)\right)S(t),\;{\dot{E}}_{1}(t)=\left(\beta_{I_{1}}I_{1}(t)+\beta_{A_{1}}A_{1}(t)\right)S(t)-(d+\alpha )E_{1}(t),\;{\dot{I}}_{1}(t)=(1-a)\alpha E_{1}(t)-(d+h+\gamma )I_{1}(t),\;{\dot{A}}_{1}(t)=a\alpha E_{1}(t)-(d+\gamma )A_{1}(t),\;\dot{H}(t)=hI_{1}(t)+hI_{2}(t)-(d+\delta +\rho )H(t),\;\dot{R}(t)=\gamma \left(I_{1}(t)+I_{2}(t)+A_{1}(t)+A_{2}(t)\right)+\rho H(t)-dR(t),\;{\dot{E}}_{2}(t)=\left(\beta_{I_{2}}I_{2}(t)+\beta_{A_{2}}A_{2}(t)\right)S(t)-(d+\alpha )E_{2}(t),\;{\dot{I}}_{2}(t)=(1-a)\alpha E_{2}(t)-(d+h+\gamma )I_{2}(t),\;{\dot{A}}_{2}(t)=a\alpha E_{2}(t)-(d+\gamma )A_{2}(t).$$

**Theorem 1.** *If the parameters of model (*3*) are positive and* Equation (2) *holds, then the solution*

$$\left.\left(S(t),E_{1}(t),I_{1}(t),A_{1}(t),H(t),R(t),E_{2}(t),I_{2}(t),A_{2}(t)\right)\right)$$

*of system (*3*) is positive and uniformly bounded on* [0, +∞).

**Proof**. We define

$$𝒯=\sup\left\{\theta >0/\forall t\in [0,\theta ],S(t)\geq 0,E_{i}(t)\geq 0,I_{i}(t)\geq 0\right.,\;\;\;\;\;\;\;\left.A_{i}(t)\geq 0,H(t)\geq 0,R(t)\geq 0,D(t)\geq 0\right\},$$

for *i* = 1, 2. Then 𝒯=+∞ Suppose that does not hold, then 𝒯<∞. Thus, by the continuity of solutions we have that

$$S(𝒯)=0,\text{ or }E_{1}(𝒯)=0,\text{ or }I_{1}(𝒯)=0,\text{ or }A_{1}(𝒯)=0,\text{ or }H(𝒯)=0,\;\text{or}$$

$$R(𝒯)=0,\text{ or }E_{2}(𝒯)=0,\text{ or }I_{2}(𝒯)=0,\text{ or }A_{2}(𝒯)=0,\text{ or }D(𝒯)=0.$$

Thus, if S(𝒯)=0, before the other variables become zero, then

$$\frac{dS(𝒯)}{dt}=\underset{t\to {𝒯}^{-}}{\lim}\frac{S(𝒯)-S(t)}{𝒯-t}\leq 0.$$

Next, from the first equation of model (3), it follows that

$$\dot{S}(𝒯)=\text{Λ}-\left(\beta_{I_{1}}I_{1}(𝒯)+\beta_{A_{1}}A_{1}(𝒯)+\beta_{I_{2}}I_{2}(𝒯)+\beta_{A_{2}}A_{2}(𝒯)\right)S(𝒯)\;\;\;\;\;-dS(𝒯)=\text{Λ}>0,$$

which is a contradiction.

Now, if $E_{1}(𝒯)=0$, above the other variables (*S*, *I*_1_, *A*_1_, *H*, *R*, *E*_2_, *I*_2_, *A*_2_, *D*) then

$$\frac{dE_{1}(𝒯)}{dt}=\underset{t\to {𝒯}^{-}}{\lim}\frac{E_{1}(𝒯)-E_{1}(t)}{𝒯-t}\leq 0,$$

and again from the second equation of system (3) one gets that

$${\dot{E}}_{1}(𝒯)=\left(\beta_{I_{1}}I_{1}(𝒯)+\beta_{A_{1}}A_{1}(𝒯)\right)S(𝒯)-(d+\alpha )E_{1}(𝒯)\;\;\;\;\;\;\;=\left(\beta_{I_{1}}I_{1}(𝒯)+\beta_{A_{1}}A_{1}(𝒯)\right)S(𝒯)>0.$$

This leads to a contradiction. In the same way, we can demonstrate similar contradictions with the other variables. As a consequence, 𝒯 could not be finite. This implies that

$$S(t)\geq 0,E_{1}(t)\geq 0,I_{1}(t)\geq 0,A_{1}(t)\geq 0,H(t)\geq 0,$$

$$R(t)\geq 0,E_{2}(t)\geq 0,I_{2}(t)\geq 0,A_{2}(t)\geq 0,D(t)\geq 0,$$

for *t* ≥ 0.

On the other hand, we can add the equations of model (3) to obtain

(4) $$\dot{N}(t)=\text{Λ}-dN(t)-\delta H(t)\leq \text{Λ}-dN(t).$$

Using the Gronwall inequalities for Equation (4) one gets

(5) $$N(t)\leq \frac{\text{Λ}}{d}+\left(N(0)-\frac{\text{Λ}}{d}\right)e^{-dt},$$

for *t* ≥ 0. Therefore, $N(t)\leq \frac{\text{Λ}}{d}$ if $N(0)\leq \frac{\text{Λ}}{d}$. Thus, the set given by

(6) $$𝓓=\left\{\left(S,E_{1},I_{1},A_{1},H,R,E_{2},I_{2},A_{2}\right)\in R_{+}^{9}/N(t)\leq \frac{\text{Λ}}{d},t\geq 0\right\},$$

is positively invariant and the solutions of model (3) remain bounded. Furthermore, if $N(0)>\frac{\text{Λ}}{d}$, then either the solution enters 𝓓 infinite time or *N*(*t*) approaches $\frac{\text{Λ}}{d}$ asymptotically. □

**Remark 1.** *This Theorem can also be proved using general techniques used for positive polynomial systems [*57,58*].*

## 3. Mathematical Stability Analysis

In this section, we find the steady state solutions, and we study their stability. First, we prove that there are two equilibrium points of interest. One equilibrium point is the disease-free and the other is the endemic. It is important to mention that this endemic point occurs when we take into account demographic factors such as births and natural deaths. Otherwise, we just obtain a disease-free equilibrium point which means that the SARS-CoV-2 virus disappears [31]. However, since we have births the system provides new susceptible individuals and this allows us to have an endemic equilibrium point. Notice that the real world situation includes births and deaths, even though the time scale of these demographic factors is slower than the one corresponding to SARS-CoV-2. We also compute the basic reproduction number ${𝓡}_{0}$ which is defined as the average number of new cases of an infection caused by one infected individual, in a population consisting of susceptible individuals only [31,76]. There are several methods to compute the basic reproduction number under different conditions and assumptions, for a nice review see [77]. Here we use the next generation matrix to compute the basic reproduction number ${𝓡}_{0}$ of the constructed mathematical model (3). It is important to remark that the effective reproduction number ${𝓡}_{t}$ is time-varying and depends on the basic reproduction number ${𝓡}_{0}$. Thus, under certain conditions (for instance only individuals in the *S*(*t*) class can get infected)${𝓡}_{t}={𝓡}_{0}S(t)/N$, which relates the value of the virus transmissibility *β* to the effective reproduction number (for more details see [31,78,79]). In addition, in this section we study the global stability of these equilibrium points using some suitable Lyapunov functionals [43,80–85].

### 3.1. Equilibrium Points and ${𝓡}_{0}$

The solutions of model (3) depend on the parameters involved in the deterministic system of differential equations for their local and global behavior. This is especially so for the basic reproduction number ${𝓡}_{0}$, which is defined as the number of secondary cases produced by an infectious individual that is introduced into the susceptible population, and in some way it makes it possible to determine the magnitude of the disease [76,86–88].

Setting the right hand side of the equations of model (3) to zero, and solving for the state variables, the disease-free equilibrium (DFE) is obtained, which is given by

(7) $$DFE=\left(S^{0},E_{1}^{0},I_{1}^{0},A_{1}^{0},H^{0},R^{0},E_{2}^{0},I_{2}^{0},A_{2}^{0}\right)=\left(\frac{\text{Λ}}{d},0,0,0,0,0,0,0,0\right).$$

Next, using the methodology of the next generation matrix [76,86], we can obtain the algebraic expression of the basic reproduction number ${𝓡}_{0}$ as the spectral radius of the $𝓕{𝒱}^{-1}$ matrix, where 𝓕 is the matrix of new infection cases and the 𝒱 matrix the of the transition terms associated with model (3), which can be written as

$$\dot{x}(t)=𝓕(x,y)-𝒱(x,y),\dot{y}(t)=G(x,y)$$

where $x(t)={\left(E_{1}(t),E_{2}(t),A_{1}(t),A_{2}(t),I_{1}(t),I_{2}(t),H(t)\right)}^{t}$, $y(t)={\left(S(t),R(t)\right)}^{t}$, and

- ${𝓕}_{i}$ is the rate of appearance of new infections in compartment *i*,
- ${𝒱}_{i}$ incorporates the remaining transitional terms, namely births, deaths, disease progression and recovery.

It is assumed that the disease-free system $\dot{y}(t)=G(0,y)$ has a unique equilibrium $y=y_{0}=\left(\frac{\text{Λ}}{d},0\right)$ that is locally asymptotically stable within the disease-free space, such that ${𝓕}_{i}(0,y)=0$, ${𝒱}_{i}(0,y)=0$, ${𝓕}_{i}(x,y)\geq 0$, ${𝒱}_{i}(x,y)\leq 0$ whenever *x*_*i*_ = 0, and $\sum_{i}{𝒱}_{i}(x,y)\geq 0$ for all *x*, *y* ≥ 0. For this case, we have

$$𝓕=\left(\begin{array}{c} \left(\beta_{I_{1}}I_{1}(t)+\beta_{A_{1}}A_{1}(t)\right)S(t)\\ \left(\beta_{I_{2}}I_{2}(t)+\beta_{A_{2}}A_{2}(t)\right)S(t)\\ 0\\ 0\\ 0\\ 0\\ 0 \end{array}\right),$$

$$𝒱=\left(\begin{array}{c} \left(d+\alpha )E_{1}(t\right)\\ \left(d+\alpha )E_{2}(t\right)\\ -(1-a)\alpha E_{1}(t)+(d+h+\gamma )I_{1}(t)\\ -(1-a)\alpha E_{2}(t)+(d+h+\gamma )I_{2}(t)\\ -a\alpha E_{1}(t)+(d+\gamma )A_{1}(t)\\ -a\alpha E_{2}(t)+(d+\gamma )A_{2}(t)\\ -hI_{1}(t)+hI_{2}(t)+(d+\delta +\rho )H(t) \end{array}\right).$$

From [76], we define two 7 × 7 matrices

$$F=\left[\frac{\partial {𝓕}_{i}\left(0,y_{0}\right)}{\partial x_{j}}\right],V=\left[\frac{\partial {𝒱}_{i}\left(0,y_{0}\right)}{\partial x_{j}}\right].$$

Thus,

$$F=\left[\begin{array}{ccccccc} 0 & 0 & \frac{\beta_{I_{1}}\text{Λ}}{d} & 0 & \frac{\beta_{A_{1}}\text{Λ}}{d} & 0 & 0\\ 0 & 0 & 0 & \frac{\beta_{I_{2}}\text{Λ}}{d} & 0 & \frac{\beta_{A_{2}}\text{Λ}}{d} & 0\\ 0 & 0 & 0 & 0 & 0 & 0 & 0\\ 0 & 0 & 0 & 0 & 0 & 0 & 0\\ 0 & 0 & 0 & 0 & 0 & 0 & 0\\ 0 & 0 & 0 & 0 & 0 & 0 & 0\\ 0 & 0 & 0 & 0 & 0 & 0 & 0 \end{array}\right]$$

and

$$V=\left[\begin{array}{ccccccc} \alpha +d & 0 & 0 & 0 & 0 & 0 & 0\\ 0 & \alpha +d & 0 & 0 & 0 & 0 & 0\\ (a-1)\alpha & 0 & V_{33} & 0 & 0 & 0 & 0\\ 0 & (a-1)\alpha & 0 & V_{44} & 0 & 0 & 0\\ -a\alpha & 0 & 0 & 0 & d+\gamma & 0 & 0\\ 0 & -a\alpha & 0 & 0 & 0 & d+\gamma & 0\\ 0 & 0 & -h & h & 0 & 0 & d+\delta +\rho \end{array}\right]$$

where **V**_33_ *= d + h + γ,* **V**_44_ *= d + h + γ*. Thus, the next generation matrix is given by

$$FV^{-1}=\left[\begin{array}{ccccccc} {𝓡}_{0_{1}} & 0 & FV_{13}^{-1} & 0 & \frac{\beta_{A_{1}}\text{Λ}}{d(d+\gamma )} & 0 & 0\\ 0 & {𝓡}_{0_{2}} & 0 & FV_{24}^{-1} & 0 & \frac{\beta_{A_{2}}\text{Λ}}{d(d+\gamma )} & 0\\ 0 & 0 & 0 & 0 & 0 & 0 & 0\\ 0 & 0 & 0 & 0 & 0 & 0 & 0\\ 0 & 0 & 0 & 0 & 0 & 0 & 0\\ 0 & 0 & 0 & 0 & 0 & 0 & 0\\ 0 & 0 & 0 & 0 & 0 & 0 & 0 \end{array}\right],$$

where $FV_{13}^{-1}=\frac{\beta_{I_{1}}\text{Λ}}{d(d+h+\gamma )}$, $FV_{24}^{-1}=\frac{\beta_{I_{2}}\text{Λ}}{d(d+h+\gamma )}$, and

(8) $${𝓡}_{0_{1}}=\frac{\text{Λ}\beta_{I_{1}}(1-a)\alpha }{d(d+\alpha )(d+h+\gamma )}+\frac{\text{Λ}\beta_{A_{1}}a\alpha }{d(d+\alpha )(d+\gamma )},\;{𝓡}_{0_{2}}=\frac{\text{Λ}\beta_{I_{2}}(1-a)\alpha }{d(d+\alpha )(d+h+\gamma )}+\frac{\text{Λ}\beta_{A_{2}}a\alpha }{d(d+\alpha )(d+\gamma )},$$

are control reproduction numbers for the two variants of SARS-CoV-2, respectively. Therefore, the spectral radius of the matrix **FV**^−1^ is given by

(9) $${𝓡}_{0}=\max\left\{{𝓡}_{0_{1}},{𝓡}_{0_{2}}\right\}.$$

### 3.2. Local Stability of Disease-Free Equilibrium Point

The basic reproduction number ${𝓡}_{0}$ threshold determines whether the disease can be eradicated or whether it will remain endemic. Thus, when ${𝓡}_{0}<1$, the transmission of the disease can be eliminated considering that the initial sizes of the subpopulations of the model (3) are in the neighborhood of attraction of the disease-free equilibrium (DFE). The following theorem guarantees the above statement.

**Theorem 2.** *The disease-free equilibrium point given in (*7*) of the model (*3*) is locally asymptotically stable if* ${𝓡}_{0}<1$, *and unstable if* ${𝓡}_{0}>1$.

**Proof.** Applying Theorem 2 given in [76], the result is confirmed. □

### 3.3. Global Stability of Disease-Free Equilibrium Point

Now, when the eradication of the disease is independent of the initial conditions of the subpopulations, then it must be shown that if ${𝓡}_{0}<1$, the disease-free equilibrium (DFE) is globally asymptotically stable (GAS). This condition is proven below.

**Theorem 3.** *The disease-free equilibrium point (*7) *of system (*3*) is globally asymptotically stable if* ${𝓡}_{0}<1$.

**Proof.** We analyze the global stability at the disease-free equilibrium point, using a suitable Lyapunov function 𝓕 as follows:

(10) $$𝓕(X(t))=E_{1}(t)+E_{2}(t)+\frac{S^{0}\beta_{I_{1}}}{d+h+\gamma }I_{1}(t)+\frac{S^{0}\beta_{I_{2}}}{d+h+\gamma }I_{2}(t)\;\;\;\;\;\;+\frac{S^{0}\beta_{A_{1}}}{d+\gamma }A_{1}(t)+\frac{S^{0}\beta_{A_{2}}}{d+\gamma }A_{2}(t),$$

where *X*(*t*) = (*S*(*t*), *E*_1_(*t*), *I*_1_(*t*), *A*_1_(*t*), *H*(*t*), *R*(*t*), *E*_2_(*t*), *I*_2_(*t*), *A*_2_(*t*)). The function 𝓕 satisfies

(11) 𝓕(DFE)=0,𝓕(X(t))>0, for all X(t)≠DFE,𝓕(X(t))→∞, when ∥X∥→∞. Thus 𝓕(X(t)) is radially unbounded.

Now, making the time derivative 𝓕(X(t))  of along the trajectories of model (3), and from (6) one gets that

$$\frac{d𝓕(X(t))}{dt}={\dot{E}}_{1}(t)+{\dot{E}}_{2}(t)+\frac{S^{0}\beta_{I_{1}}}{d+h+\gamma }{\dot{I}}_{1}(t)\;\;\;\;\;\;+\frac{S^{0}\beta_{I_{2}}}{d+h+\gamma }{\dot{I}}_{2}(t)+\frac{S^{0}\beta_{A_{1}}}{d+\gamma }{\dot{A}}_{1}(t)+\frac{S^{0}\beta_{A_{2}}}{d+\gamma }{\dot{A}}_{2}(t)\;\;\;\;\;\;\;=\left(\beta_{I_{1}}I_{1}(t)+\beta_{A_{1}}A_{1}(t)\right)S(t)-(d+\alpha )E_{1}(t)\;\;\;\;\;\;+\left(\beta_{I_{2}}I_{2}(t)+\beta_{A_{2}}A_{2}(t)\right)S(t)-(d+\alpha )E_{2}(t)\;\;\;\;\;\;+\frac{S^{0}\beta_{I_{1}}(1-a)\alpha }{d+h+\gamma }E_{1}(t)-S^{0}\beta_{I_{1}}I_{1}(t)\;\;\;\;\;\;+\frac{S^{0}\beta_{I_{2}}\left(1-a_{2}\right)\alpha }{d+h+\gamma }E_{2}(t)-S^{0}\beta_{I_{2}}I_{2}(t)\;\;\;\;\;\;+\frac{S^{0}\beta_{A_{1}}a\alpha }{d+\gamma }E_{1}(t)-S^{0}\beta_{A_{1}}A_{1}(t)+\frac{S^{0}\beta_{A_{2}}a\alpha }{d+\gamma }E_{2}(t)-S^{0}\beta_{A_{2}}A_{2}(t)\;\;\;\;\;\;\;\leq (d+\alpha )\left(\frac{S^{0}\beta_{I_{1}}(1-a)\alpha }{\left(d+\alpha )(d+h+\gamma \right)}+\frac{S^{0}\beta_{A_{1}}a\alpha }{\left(d+\alpha )(d+\gamma \right)}-1\right)E_{1}(t)\;\;\;\;\;\;+(d+\alpha )\left(\frac{S^{0}\beta_{I_{2}}\left(1-a_{2}\right)\alpha }{\left(d+\alpha )(d+h+\gamma \right)}+\frac{S^{0}\beta_{A_{2}}a\alpha }{\left(d+\alpha )(d+\gamma \right)}-1\right)E_{2}(t)\;\;\;\;\;\;\;=(d+\alpha )\left({𝓡}_{0_{1}}-1\right)E_{1}(t)+(d+\alpha )\left({𝓡}_{0_{2}}-1\right)E_{2}(t)\;\;\;\;\;\;\;\leq (d+\alpha )\left({𝓡}_{0}-1\right)\left(E_{1}(t)+E_{2}(t)\right).$$

Thus, $\frac{d𝓕\left(X\left(t\right)\right)}{dt}\leq 0$ when ${𝓡}_{0}\leq 1$, and $\frac{d𝓕\left(X\left(t\right)\right)}{dt}=0$ if and only if *E*_1_(*t*) = 0 and *E*_2_(*t*) = 0. This implies that the set

$${ℒ}_{DFE}=\left\{X(t)\in 𝓓:\frac{d𝓕(X(t))}{dt}=0\right\}$$

is reduced to {*DFE*}. Then, applying LaSalle’s Invariance Principle [89], it follows that if ${𝓡}_{0}\leq 1$, the solutions of (3) tend to *DFE* and thus the disease free equilibrium point is globally stable in 𝓓 □

### 3.4. Global Stability of New SARS-CoV-2 Variant Endemic Point

The determination of endemic equilibrium points in a disease is important because it allows health institutions and governments to take preventive measures to control the level of transmission and prevent it from becoming endemic. We are interested in analyzing the behavior of the solutions of model (3) when the transmission rate of the new SARS-CoV-2 is higher than that of the preexistent one, that is, $\beta_{I_{2}}>\beta_{I_{1}}$, and $\beta_{A_{2}}>\beta_{A_{1}}$. These transmission rates measure in some way the effect of the magnitude of the second strain with respect to the the first one through time. Thus, the endemic equilibrium can be found by setting the right hand side of the equations of the model (3) to zero, that is,

(12) $$0=\text{Λ}-dS^{*}-\left(\beta_{I_{1}}I_{1}^{*}+\beta_{A_{1}}A_{1}^{*}+\beta_{I_{2}}I_{2}^{*}+\beta_{A_{2}}A_{2}^{*}\right)S^{*},\;0=\left(\beta_{I_{1}}I_{1}^{*}+\beta_{A_{1}}A_{1}^{*}\right)S^{*}-(d+\alpha )E_{1}^{*},\;0=(1-a)\alpha E_{1}^{*}-(d+h+\gamma )I_{1}^{*},\;0=a\alpha E_{1}^{*}-(d+\gamma )A_{1}^{*},\;0=hI_{1}^{*}+hI_{2}^{*}-(d+\delta +\rho )H^{*},\;0=\gamma \left(I_{1}^{*}+A_{1}^{*}+I_{2}^{*}+A_{2}^{*}\right)+\rho H^{*}-dR^{*},\;0=\left(\beta_{I_{2}}I_{2}^{*}+\beta_{A_{2}}A_{2}^{*}\right)S^{*}-(d+\alpha )E_{2}^{*},\;0=(1-a)\alpha E_{2}^{*}-(d+h+\gamma )I_{2}^{*},\;0=a\alpha E_{2}^{*}-(d+\gamma )A_{2}^{*}.$$

It is clear from the first equation of system (12) that $S^{*}>0$. Moreover, $\Lambda -dS^{*}>0$, which implies that $S^{*}\in 𝓓$. Now, we need the following proposition to get the endemic point.

**Proposition 1.** *When $\beta_{I_{2}}>\beta_{I_{1}}$*, *and $\beta_{A_{2}}>\beta_{A_{1}}$*, *then $E_{1}^{*}=I_{1}^{*}=A_{1}^{*}=0$*.

**Proof.** Suppose that $E_{1}^{*}=I_{1}^{*}=A_{1}^{*}=0$, does not hold. Hence, if any of the points $E_{1}^{*}$, $I_{1}^{*}$, $A_{1}^{*}$ is zero, a contradiction follows from (12). Suppose the case when $E_{1}^{*}>0$, $I_{1}^{*}>0$, $A_{1}^{*}>0$, then from (12) we have that

$$I_{2}^{*}=I_{1}^{*}\frac{E_{2}^{*}}{E_{1}^{*}},A_{2}^{*}=A_{1}^{*}\frac{E_{2}^{*}}{E_{1}^{*}}\text{. }$$

Using the seventh equation of system (12), one gets that

(13) $$(\alpha +d)E_{2}^{*}=\left(\beta_{I_{2}}I_{2}^{*}+\beta_{A_{2}}A_{2}^{*}\right)S^{*}>\left(\beta_{I_{1}}I_{2}^{*}+\beta_{A_{1}}A_{2}^{*}\right)S^{*}\;\;\;\;\;\;\;=\left(\beta_{I_{2}}I_{1}^{*}\frac{E_{2}^{*}}{E_{1}^{*}}+\beta_{A_{2}}A_{1}^{*}\frac{E_{2}^{*}}{E_{1}^{*}}\right)S^{*},$$

which is a contradiction with the second equation of system (12). □

Therefore, Proposition 1 allows us to affirm that the only endemic point given by

(14) $$S_{2}EP=\left(S_{2}^{*},0,0,0,H_{2}^{*},R_{2}^{*},E_{2,2}^{*},I_{2,2}^{*},A_{2,2}^{*}\right),$$

where

(15) $$0=\text{Λ}-dS_{2}^{*}-\left(\beta_{I_{2}}I_{2,2}^{*}+\beta_{A_{2}}A_{2,2}^{*}\right)S_{2}^{*},\;0=hI_{2,2}^{*}-(d+\delta +\rho )H_{2}^{*},\;0=\gamma \left(I_{2,2}^{*}+A_{2,2}^{*}\right)+\rho H_{2}^{*}-dR_{2}^{*},\;0=\left(\beta_{I_{2}}I_{2,2}^{*}+\beta_{A_{2}}A_{2,2}^{*}\right)S_{2}^{*}-(d+\alpha )E_{2,2}^{*},\;0=\left(1-a_{2}\right)\alpha E_{2,2}^{*}-(d+h+\gamma )I_{2,2}^{*},\;0=a_{2}\alpha E_{2,2}^{*}-(d+\gamma )A_{2,2}^{*}.$$

After performing some algebraic manipulations in the system (15), it follows that the point *S*_2_*EP* satisfies

(16) $$S_{2}^{*}=\frac{\text{Λ}}{d{𝓡}_{0_{2}}},\;E_{2,2}^{*}=\frac{\text{Λ}}{d+\alpha }\frac{{𝓡}_{0_{2}}-1}{{𝓡}_{0_{2}}},\;A_{2,2}^{*}=\frac{a\alpha \text{Λ}}{\left(d+\gamma )(d+\alpha \right)}\frac{{𝓡}_{0_{2}}-1}{{𝓡}_{0_{2}}},\;I_{2,2}^{*}=\frac{(1-a)\alpha \text{Λ}}{\left(d+h+\gamma )(d+\alpha \right)}\frac{{𝓡}_{0_{2}}-1}{{𝓡}_{0_{2}}},\;H_{2}^{*}=\frac{h(1-a)\alpha \text{Λ}}{\left(d+\delta +\rho )(d+h+\gamma )(d+\alpha \right)}\frac{{𝓡}_{0_{2}}-1}{{𝓡}_{0_{2}}},\;R_{2}^{*}=\left(\frac{\gamma (1-a)\alpha \text{Λ}}{d(d+h+\gamma )(d+\alpha )}+\frac{\gamma a\alpha \text{Λ}}{d(d+\gamma )(d+\alpha )}\right.\;\left.+\frac{\rho }{d}\frac{h(1-a)\alpha \text{Λ}}{\left(d+\delta +\rho )(d+h+\gamma )(d+\alpha \right)}\right)\frac{{𝓡}_{0_{2}}-1}{{𝓡}_{0_{2}}}.$$

We can abbreviate the above results in the following proposition.

**Proposition 2.** *The endemic point S*_2_*EP given by (14) exists if ${𝓡}_{0_{2}}>\max\left\{{𝓡}_{0_{1}},1\right\}$*.

In the construction of the Lyapunov function to analyze the global stability of the equilibrium point (14), we use the Volterra function,

G(w)=w−1−lnw,

which is non-negative for w>0 and $G\left(w\right)=0$ if and only if w=1.

**Theorem 4.** *When* ${𝓡}_{0_{2}}>1>{𝓡}_{0_{1}}$, *the endemic equilibrium point S*_2_*EP given by (*14*) is globally asymptotically stable on* 𝓓.

**Proof.** Let fg $ℒ\left(X\left(t\right)\right)$ be the Lyapunov function given by

(17) $$ℒ(X(t))=E_{1}(t)+\frac{S^{0}\beta_{I_{1}}}{d+h+\gamma }I_{1}(t)+\frac{S^{0}\beta_{A_{1}}}{d+\gamma }A_{1}(t)\;\;\;\;\;\;\;+S_{2}^{*}\left(\frac{S(t)}{S_{2}^{*}}-1-\ln\left(\frac{S(t)}{S_{2}^{*}}\right)\right)+E_{2,2}^{*}\left(\frac{E_{2}(t)}{E_{2,2}^{*}}-1-\ln\left(\frac{E_{2}(t)}{E_{2,2}^{*}}\right)\right)\;\;\;\;\;\;\;+\frac{\beta_{I_{2}}I_{2,2}^{*}S_{2}^{*}}{(1-a)\alpha E_{2,2}^{*}}I_{2,2}^{*}\left(\frac{I_{2}(t)}{I_{2,2}^{*}}-1-\ln\left(\frac{I_{2}(t)}{I_{2,2}^{*}}\right)\right)\;\;\;\;\;\;\;+\frac{\beta_{A_{2}}A_{2,2}^{*}S_{2}^{*}}{a\alpha E_{2,2}^{*}}A_{2,2}^{*}\left(\frac{A_{2}(t)}{A_{2,2}^{*}}-1-\ln\left(\frac{A_{2}(t)}{A_{2,2}^{*}}\right)\right),$$

with *X*(*t*) = (*S*(*t*), *E*_1_(*t*), *I*_1_(*t*), *A*_1_(*t*), *H*(*t*), *R*(*t*), *E*_2_(*t*), *I*_2_(*t*), *A*_2_(*t*)). It is clear that the function ℒ satisfies (11). Next, making the time derivative of $ℒ\left(X\left(t\right)\right)$ along the trajectories of model (3), one gets that

(18) $$\frac{dℒ(X(t))}{dt}={\dot{E}}_{1}(t)+\frac{S^{0}\beta_{I_{1}}}{d+h+\gamma }{\dot{I}}_{1}(t)+\frac{S^{0}\beta_{A_{1}}}{d+\gamma }{\dot{A}}_{1}(t)\;\;\;\;\;\;\;+\left(1-\frac{S_{2}^{*}}{S(t)}\right)\dot{S}(t)+\left(1-\frac{E_{2,2}^{*}}{E_{2}(t)}\right){\dot{E}}_{2}(t)\;\;\;\;\;\;\;+\frac{\beta_{I_{2}}I_{2,2}^{*}S_{2}^{*}}{(1-a)\alpha E_{2,2}^{*}}\left(1-\frac{I_{2,2}^{*}}{I_{2}(t)}\right){\dot{I}}_{2}(t)\;\;\;\;\;\;\;+\frac{\beta_{A_{2}}A_{2,2}^{*}S_{2}^{*}}{a\alpha E_{2,2}^{*}}\left(1-\frac{A_{2,2}^{*}}{A_{2}(t)}\right){\dot{A}}_{2}(t).$$

Replacing the derivatives of the state variables in the above expression, we get

(19) $$\frac{dℒ(X(t))}{dt}=\left(\beta_{I_{1}}I_{1}(t)+\beta_{A_{1}}A_{1}(t)\right)S(t)-(d+\alpha )E_{1}(t)\;\;\;\;\;\;\;+\frac{S^{0}\beta_{I_{1}}}{d+h+\gamma }\left(\left(1-a)\alpha E_{1}(t)-(d+h+\gamma )I_{1}(t\right)\right)\;\;\;\;\;\;\;+\frac{S^{0}\beta_{A_{1}}}{d+\gamma }\left(a\alpha E_{1}(t)-(d+\gamma )A_{1}(t)\right)\;\;\;\;\;\;\;+\left(1-\frac{S_{2}^{*}}{S(t)}\right)(\text{Λ}-dS(t)\;\;\;\;\;\;\;\left.-\left(\beta_{I_{1}}I_{1}(t)+\beta_{A_{1}}A_{1}(t)+\beta_{I_{2}}I_{2}(t)+\beta_{A_{2}}A_{2}(t)\right)S(t)\right)\;\;\;\;\;\;\;+\left(1-\frac{E_{2,2}^{*}}{E_{2}(t)}\right)\left(\left(\beta_{I_{2}}I_{2}(t)+\beta_{A_{2}}A_{2}(t)\right)S(t)-(d+\alpha )E_{2}(t)\right)\;\;\;\;\;\;\;+\frac{\beta_{I_{2}}I_{2,2}^{*}S_{2}^{*}}{(1-a)\alpha E_{2,2}^{*}}\left(1-\frac{I_{2,2}^{*}}{I_{2}(t)}\right)\left(\left(1-a_{2}\right)\alpha E_{2}(t)-(d+h+\gamma )I_{2}(t)\right)\;\;\;\;\;\;\;+\frac{\beta_{A_{2}}A_{2,2}^{*}S_{2}^{*}}{a\alpha E_{2,2}^{*}}\left(1-\frac{A_{2,2}^{*}}{A_{2}(t)}\right)\left(a_{2}\alpha E_{2}(t)-(d+\gamma )A_{2}(t)\right).$$

From the equations given in (15), we have that

(20) $$\text{Λ}=dS_{2}^{*}+\left(\beta_{I_{2}}I_{2,2}^{*}+\beta_{A_{2}}A_{2,2}^{*}\right)S_{2}^{*},d+\alpha =\frac{\left(\beta_{I_{2}}I_{2,2}^{*}+\beta_{A_{2}}A_{2,2}^{*}\right)S_{2}^{*}}{E_{2,2}^{*}}\;d+h+\gamma =\frac{\left(1-a_{2}\right)\alpha E_{2,2}^{*}}{I_{2,2}^{*}},d+\gamma =\frac{a_{2}\alpha E_{2,2}^{*}}{A_{2,2}^{*}}.$$

Substituting (20) in (19) and regrouping terms it follows that

(21) $$\frac{dℒ(X(t))}{dt}=-\frac{d{\left(S(t)-S_{2}^{*}\right)}^{2}}{S(t)}+3\beta_{I_{2}}I_{2,2}^{*}S_{2}^{*}+3\beta_{A_{2}}A_{2,2}^{*}S_{2}^{*}+\left(\beta_{I_{1}}I_{1}(t)+\beta_{A_{1}}A_{1}(t)\right)S_{2}^{*}\;\;\;\;\;\;-S^{0}\beta_{I_{1}}I_{1}(t)-S^{0}\beta_{A_{1}}A_{1}(t)+(d+\alpha )E_{1}(t)\left({𝓡}_{0_{1}}-1\right)\;\;\;\;\;\;-\frac{\beta_{I_{2}}I_{2,2}^{*}S_{2}^{*}S_{2}^{*}}{S(t)}-\frac{\beta_{A_{2}}A_{2,2}^{*}S_{2}^{*}S_{2}^{*}}{S(t)}-\frac{\beta_{I_{2}}E_{2,2}^{*}I_{2}(t)S(t)}{E_{2}(t)}-\frac{\beta_{A_{2}}E_{2,2}^{*}A_{2}(t)S(t)}{E_{2}(t)}\;\;\;\;\;\;-\frac{\beta_{I_{2}}I_{2,2}^{*}I_{2,2}^{*}S_{2}^{*}E_{2}(t)}{I_{2}(t)E_{2,2}^{*}}-\frac{\beta_{A_{2}}A_{2,2}^{*}A_{2,2}^{*}S_{2}^{*}E_{2}(t)}{A_{2}(t)E_{2,2}^{*}}.$$

Thus,

(22) $$\frac{dℒ(X(t))}{dt}\leq -\frac{d{\left(S(t)-S_{2}^{*}\right)}^{2}}{S(t)}\;\;\;\;\;\;\;+\beta_{I_{2}}I_{2,2}^{*}S_{2}^{*}\left(3-\frac{S_{2}^{*}}{S(t)}-\frac{E_{2,2}^{*}I_{2}(t)S(t)}{E_{2}(t)I_{2,2}^{*}S_{2}^{*}}-\frac{I_{2,2}^{*}E_{2}(t)}{I_{2}(t)E_{2,2}^{*}}\right)\;\;\;\;\;\;\;+\beta_{A_{2}}A_{2,2}^{*}S_{2}^{*}\left(3-\frac{S_{2}^{*}}{S(t)}-\frac{E_{2,2}^{*}A_{2}(t)S(t)}{E_{2}(t)A_{2,2}^{*}S_{2}^{*}}-\frac{A_{2,2}^{*}E_{2}(t)}{A_{2}(t)E_{2,2}^{*}}\right)\;\;\;\;\;\;\;+(d+\alpha )E_{1}(t)\left({𝓡}_{0_{1}}-1\right).$$

Using the relationship between arithmetic and geometric means, one gets that

(23) $$\left(3-\frac{S_{2}^{*}}{S(t)}-\frac{E_{2,2}^{*}I_{2}(t)S(t)}{E_{2}(t)I_{2,2}^{*}S_{2}^{*}}-\frac{I_{2,2}^{*}E_{2}(t)}{I_{2}(t)E_{2,2}^{*}}\right)\leq 0,\;\left(3-\frac{S_{2}^{*}}{S(t)}-\frac{E_{2,2}^{*}A_{2}(t)S(t)}{E_{2}(t)A_{2,2}^{*}S_{2}^{*}}-\frac{A_{2,2}^{*}E_{2}(t)}{A_{2}(t)E_{2,2}^{*}}\right)\leq 0.$$

Therefore, if ${𝓡}_{0_{2}}>1>{𝓡}_{0_{1}}$ then $\frac{dℒ(X(t))}{dt}\leq 0$. Moreover, $\frac{dℒ(X(t))}{dt}=0$ if only if $E_{1}(t)=I_{1}(t)=A_{1}(t)=0$, $S(t)=S_{2}^{*}$, $A_{2}(t)=A_{2,2}^{*}$, $I_{2}(t)=I_{2,2}^{*}$. Thus, using LaSalle’s principle theorem [89], the set defined as

(24) $${ℒ}_{S_{2}EP}=\left\{X(t)\in 𝓓:\frac{dℒ(X(t))}{dt}=0\right\}=\left\{S_{2}EP\right\},$$

is invariant and contains the single point *S*_2_*EP*. Therefore, the endemic equilibrium given by (14) is said to be globally asymptotically stable in the region 𝓓 if ${𝓡}_{0_{2}}>1>{𝓡}_{0_{1}}$. □

**Theorem 5.** *When ${𝓡}_{0_{2}}>{𝓡}_{0_{1}}>1$*, *the endemic equilibrium point S*_2_*EP given by (14) is globally asymptotically stable on* 𝓓.

**Proof.** In this case, we use the following Lyapunov function

(25) $$S(X(t))={𝓡}_{0_{2}}S_{2}^{*}\left(\frac{S(t)}{S_{2}^{*}}-1-\ln\left(\frac{S(t)}{S_{2}^{*}}\right)\right)+{𝓡}_{0_{2}}E_{1}(t)\;\;\;\;\;\;\;+\frac{S^{0}\beta_{I_{1}}}{d+h+\gamma }I_{1}(t)+\frac{S^{0}\beta_{A_{1}}}{d+\gamma }A_{1}(t)\;\;\;\;\;\;\;+{𝓡}_{0_{2}}E_{2,2}^{*}\left(\frac{E_{2}(t)}{E_{2,2}^{*}}-1-\ln\left(\frac{E_{2}(t)}{E_{2,2}^{*}}\right)\right)\;\;\;\;\;\;\;+\frac{{𝓡}_{0_{2}}\beta_{I_{2}}I_{2,2}^{*}S_{2}^{*}}{(1-a)\alpha E_{2,2}^{*}}I_{2,2}^{*}\left(\frac{I_{2}(t)}{I_{2,2}^{*}}-1-\ln\left(\frac{I_{2}(t)}{I_{2,2}^{*}}\right)\right)\;\;\;\;\;\;\;+\frac{{𝓡}_{0_{2}}\beta_{A_{2}}A_{2,2}^{*}S_{2}^{*}}{a\alpha E_{2,2}^{*}}A_{2,2}^{*}\left(\frac{A_{2}(t)}{A_{2,2}^{*}}-1-\ln\left(\frac{A_{2}(t)}{A_{2,2}^{*}}\right)\right),$$

where *X*(*t*) = (*S*(*t*), *E*_1_(*t*), *I*_1_(*t*), *A*_1_(*t*), *H*(*t*), *R*(*t*), *E*_2_(*t*), *I*_2_(*t*), *A*_2_(*t*)). Again the function ℒ holds (11). Now, taking the time derivative of S(X(t)) along the trajectories of system (3), we obtain

(26) $$\frac{dℒ(X(t))}{dt}={𝓡}_{0_{2}}\left(1-\frac{S_{2}^{*}}{S(t)}\right)\dot{S}(t)+{𝓡}_{0_{2}}{\dot{E}}_{1}(t)+\frac{S^{0}\beta_{I_{1}}}{d+h+\gamma }{\dot{I}}_{1}(t)\;\;\;\;\;\;\;+\frac{S^{0}\beta_{A_{1}}}{d+\gamma }{\dot{A}}_{1}(t)+{𝓡}_{0_{2}}\left(1-\frac{E_{2,2}^{*}}{E_{2}(t)}\right){\dot{E}}_{2}(t)\;\;\;\;\;\;\;+\frac{{𝓡}_{0_{2}}\beta_{I_{2}}I_{2,2}^{*}S_{2}^{*}}{(1-a)\alpha E_{2,2}^{*}}\left(1-\frac{I_{2,2}^{*}}{I_{2}(t)}\right){\dot{I}}_{2}(t)\;\;\;\;\;\;\;+\frac{{𝓡}_{0_{2}}\beta_{A_{2}}A_{2,2}^{*}S_{2}^{*}}{a\alpha E_{2,2}^{*}}\left(1-\frac{A_{2,2}^{*}}{A_{2}(t)}\right){\dot{A}}_{2}(t).$$

Substituting the derivatives of the state variables, using (20) and regrouping terms, we get that

(27) $$\frac{dℒ(X(t))}{dt}=-\frac{d{𝓡}_{0_{2}}{\left(S(t)-S_{2}^{*}\right)}^{2}}{S(t)}+3{𝓡}_{0_{2}}\beta_{I_{2}}I_{2,2}^{*}S_{2}^{*}+3{𝓡}_{0_{2}}\beta_{A_{2}}A_{2,2}^{*}S_{2}^{*}\;\;\;\;\;\;\;+(d+\alpha )E_{1}(t)\left({𝓡}_{0_{1}}-{𝓡}_{0_{2}}\right)\;\;\;\;\;\;\;-\frac{{𝓡}_{0_{2}}\beta_{I_{2}}I_{2,2}^{*}S_{2}^{*}S_{2}^{*}}{S(t)}-\frac{{𝓡}_{0_{2}}\beta_{A_{2}}A_{2,2}^{*}S_{2}^{*}S_{2}^{*}}{S(t)}-\frac{{𝓡}_{0_{2}}\beta_{I_{2}}E_{2,2}^{*}I_{2}(t)S(t)}{E_{2}(t)}\;\;\;\;\;\;\;-\frac{{𝓡}_{0_{2}}\beta_{A_{2}}E_{2,2}^{*}A_{2}(t)S(t)}{E_{2}(t)}\;\;\;\;\;\;\;-\frac{{𝓡}_{0_{2}}\beta_{I_{2}}I_{2,2}^{*}I_{2,2}^{*}S_{2}^{*}E_{2}(t)}{I_{2}(t)E_{2,2}^{*}}-\frac{{𝓡}_{0_{2}}\beta_{A_{2}}A_{2,2}^{*}A_{2,2}^{*}S_{2}^{*}E_{2}(t)}{A_{2}(t)E_{2,2}^{*}}.$$

Thus,

(28) $$\frac{dℒ(X(t))}{dt}=-\frac{{𝓡}_{0_{2}}d{\left(S(t)-S_{2}^{*}\right)}^{2}}{S(t)}+(d+\alpha )E_{1}(t)\left({𝓡}_{0_{1}}-{𝓡}_{0_{2}}\right)\;\;\;\;\;\;\;+{𝓡}_{0_{2}}\beta_{I_{2}}I_{2,2}^{*}S_{2}^{*}\left(3-\frac{S_{2}^{*}}{S(t)}-\frac{E_{2,2}^{*}I_{2}(t)S(t)}{E_{2}(t)I_{2,2}^{*}S_{2}^{*}}-\frac{I_{2,2}^{*}E_{2}(t)}{I_{2}(t)E_{2,2}^{*}}\right)\;\;\;\;\;\;\;+{𝓡}_{0_{2}}\beta_{A_{2}}A_{2,2}^{*}S_{2}^{*}\left(3-\frac{S_{2}^{*}}{S(t)}-\frac{E_{2,2}^{*}A_{2}(t)S(t)}{E_{2}(t)A_{2,2}^{*}S_{2}^{*}}-\frac{A_{2,2}^{*}E_{2}(t)}{A_{2}(t)E_{2,2}^{*}}\right).$$

Therefore, from (23) and if ${𝓡}_{0_{2}}>{𝓡}_{0_{1}}>1$, then $\frac{dℒ(X(t))}{dt}\leq 0$. Moreover, $\frac{dℒ(X(t))}{dt}=0$ if only if $E_{1}(t)=I_{1}(t)=A_{1}(t)=0$, $S(t)=S_{2}^{*}$, $A_{2}(t)=A_{2,2}^{*}$, $I_{2}(t)=I_{2,2}^{*}$, that is, (24) holds. Thus, again by the LaSalle’s principle theorem, the Strain-2 Endemic Equilibrium point *S*_2_*EP* given by (16) is globally asymptotically stable on 𝓓, provided that ${𝓡}_{0_{2}}>{𝓡}_{0_{1}}>1$. □

## 4. Numerical Simulation Results

In this section, we will perform some numerical simulations of the mathematical model (1) in order to corroborate the previous theoretical results. These simulations will allow us to analyze only qualitatively the potential impact of a new SARS-CoV-2 variant with a higher transmission rate than that of the preexistent variant. The numerical simulations presented here are not a prediction or forecast of the COVID-19 pandemic, even though we used parameter values from the scientific literature. The results presented here allow us to have a better understanding of the introduction of a new SARS-CoV-2 variant with a higher transmission rate.

The numerical simulations are performed using the parameter values given in Table 1. The initial conditions for the subpopulations are modified in order to corroborate the local and global theoretical stability results. Some of these previous values are based approximately on the demographics and the current situation in Colombia. We include several numerical simulations with different transmission rates of the two SARS-CoV-2 variants. Two important key parameters are the transmissibility of the two SARS-CoV-2 variants, since they are related to the basic reproduction number ${𝓡}_{0}$ [30,31,76,86], and therefore to the effective reproduction number ${𝓡}_{t}$ [79]. The scientific literature provides a wide range for these parameter values, but some studies have mentioned that the new SARS-CoV-2 variant (VOC-202012/01) is in the range of 20–70% more transmissible [1,23,27,90]. The numerical simulations provide the steady states that include values for the different subpopulations, such as the number of infected people with each variant, susceptible individuals, hospitalized, and deaths. These metrics are useful and relevant to health authorities and people in general.

In the numerical simulations, we are interested in the situation in which a new more transmissible SARS-CoV-2 variant is introduced ($\beta_{I_{2}}>\beta_{I_{1}}$ and $\beta_{A_{2}}>\beta_{A_{1}}$). We can assume that this new variant is the VOC-202012/01 of lineage B.1.1.7. However, the mathematical model considers that it could be another SARS-CoV-2 variant, but more transmissible. Therefore, this study is also valid, for instance, if the new second variant is the 501Y.V2 [91]. We assume that the rates of virus transmission in asymptomatic and symptomatic individuals are time-invariant from the beginning of the simulation. This implies that people have approximately the same behavior (on average) regarding SARS-CoV-2 virus spread protection. This assumption is plausible in reality but it might change if the number of cases and deaths increase dramatically due to the new SARS-CoV-2 variant. In countries where a vaccination program is advancing quickly it is necessary to construct an alternative model with more classes and parameters, among which is the vaccinated class. For example, in previous work, both discrete and continuous mathematical models have included several subclasses of the vaccinated class where the individuals have less probability of getting infected, transmitting the virus or dying [52,65]. If some changes, such as lock-downs or curfews, occur then the mathematical model (1) should include time-varying parameters, which for instance have been used to study influenza [38]. This would make the mathematical model more complex due to time-varying parameters. We assume that the parameters related to the latent and infectiousness stages are the same for both SARS-CoV-2 variants. For the death rate of hospitalized individuals we use data from different scientific sources, but since we are not forecasting it is not crucial in this study [50,65,92–94]. For the asymptomatic proportion we chose, as a conservative starting point, the percentage of infections that are asymptomatic as 50% [1]. The available data from scientific papers and health institutions have a great variation [1,50,72,95–100]. For the numerical simulations we additionally considered percentages varying between 30–70% [1,72]. For the initial conditions, in all the numerical simulations we compute the initial susceptible subpopulation using the fact that *S*(0) = *N*(0) – *E*_1_(0) – *I*_1_(0) – *A*_1_(0) – *E*_2_(0) – *I*_2_(0) – *A*_2_(0) – *R*(0) – *H*(0).

**Figure 2.**
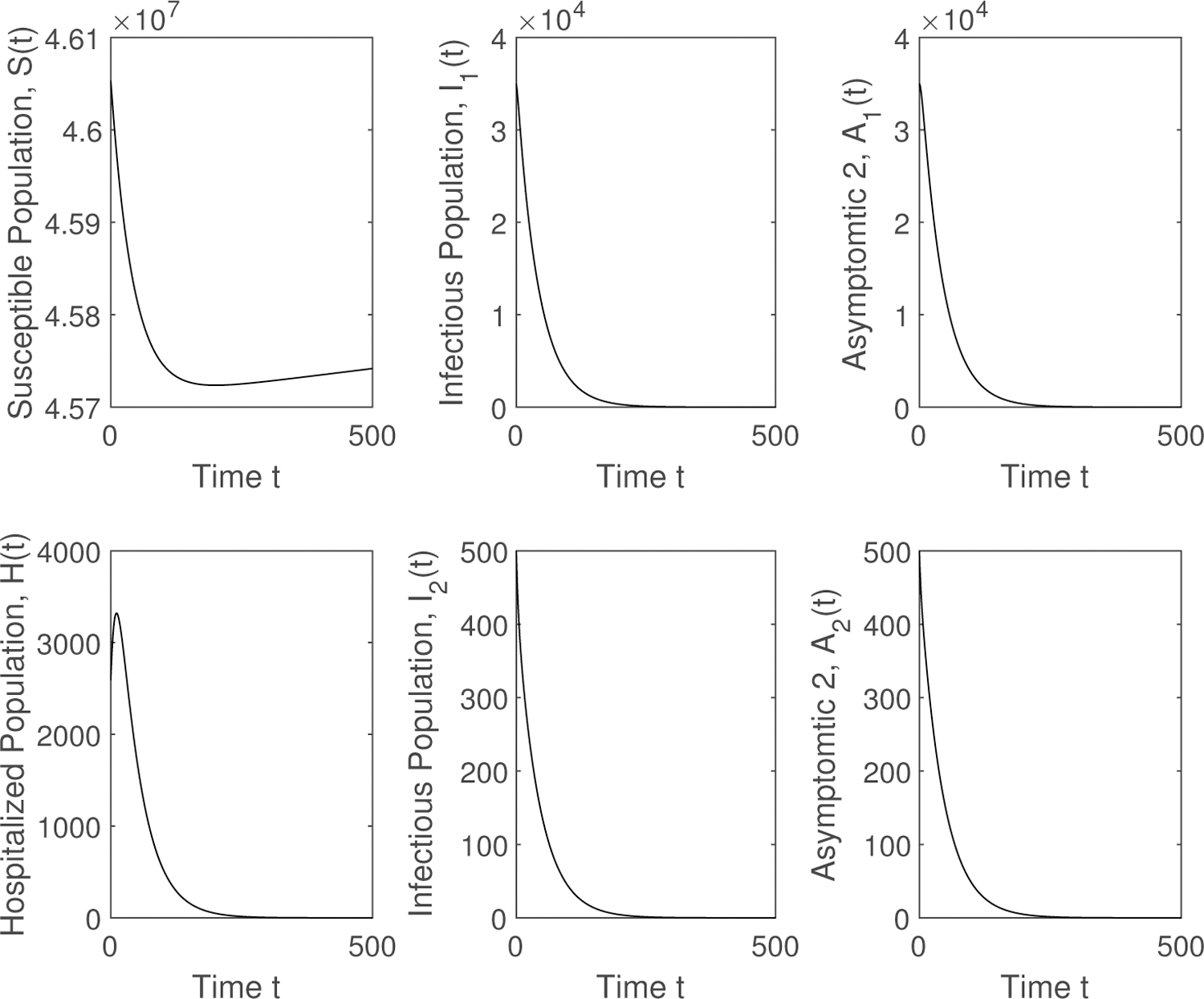
Numerical simulation of the mathematical model (1) when ${𝓡}_{0_{1}}<1$ and ${𝓡}_{0_{2}}<1$. The SARS-CoV2 variants disappear and the system reaches the disease free equilibrium point.

**Figure 3.**
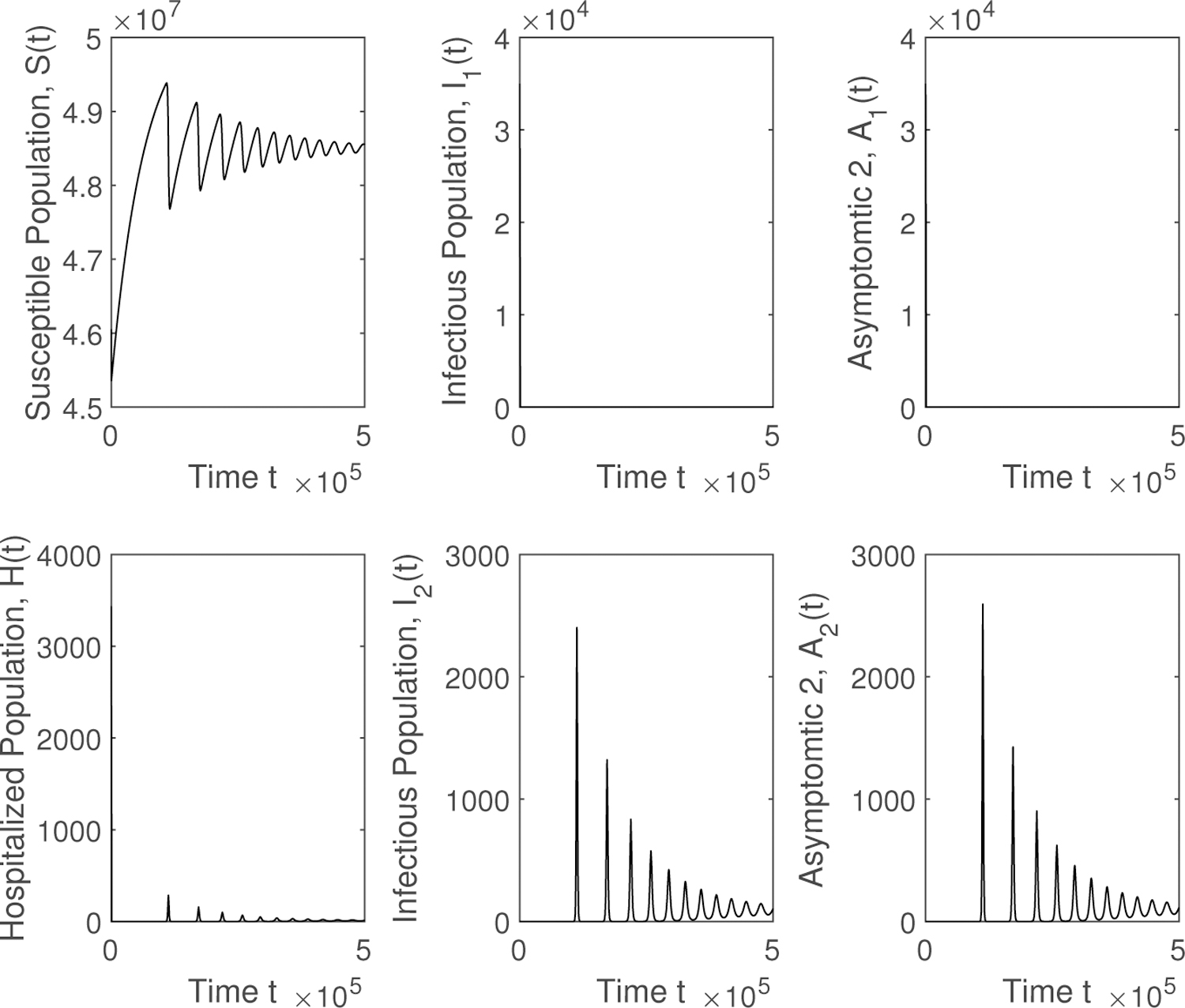
Numerical simulation of the mathematical model (1) when ${𝓡}_{0_{1}}<1$ and ${𝓡}_{0_{2}}>1$. The new highly transmissible SARS-CoV-2 variant dominates the preexistent variant, which disappears and the system reaches the endemic equilibrium point *EP*.

**Figure 4.**
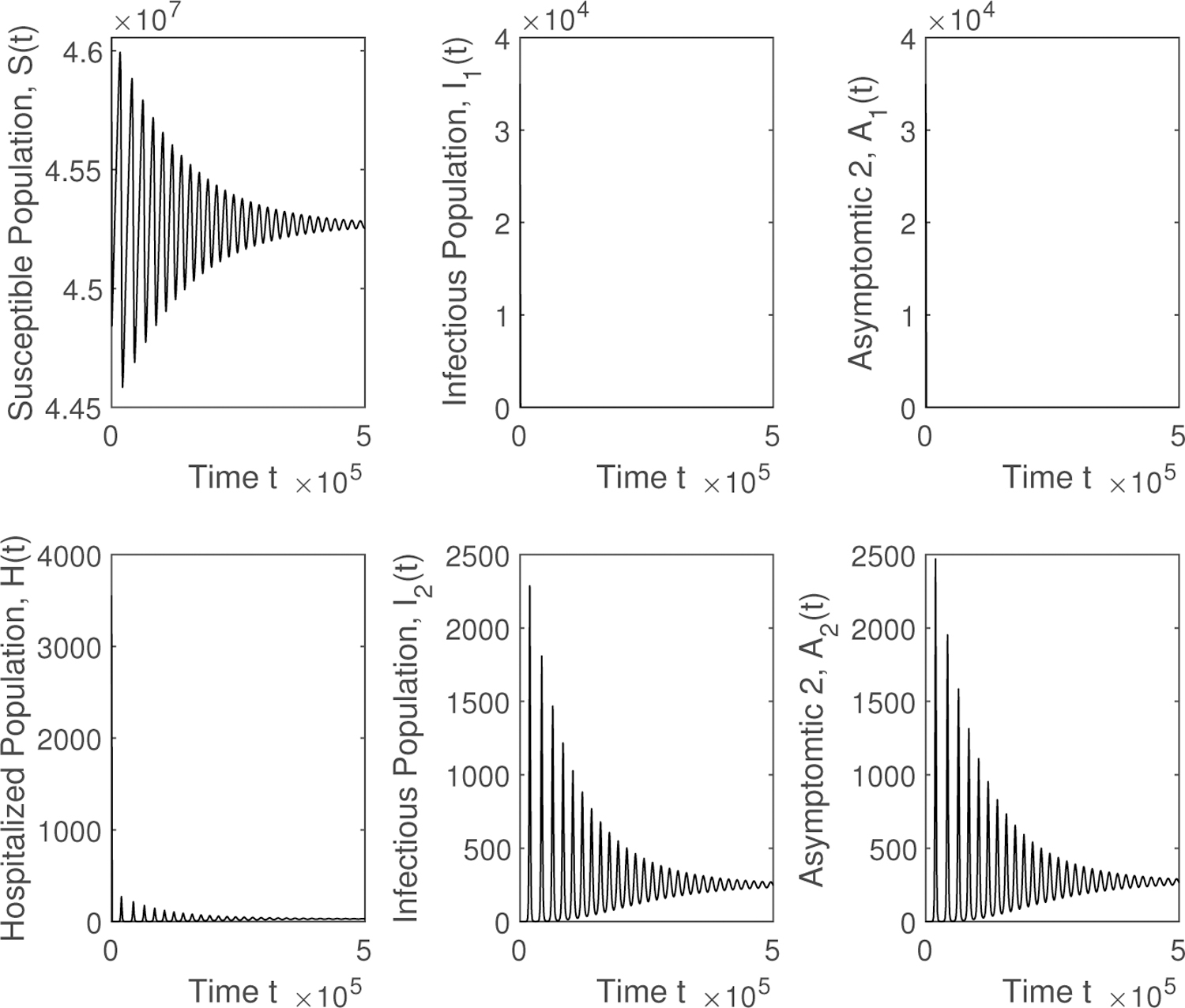
Numerical simulation of the mathematical model (1) when ${𝓡}_{0_{2}}>{𝓡}_{0_{1}}>1$. The new highly transmissible SARS-CoV-2 variant still dominates the preexistent variant, which disappears even though ${𝓡}_{0_{1}}>1$. The system reaches the endemic equilibrium point *EP*.

Finally, we show the qualitative results in a graphic form for different scenarios, varying the contagiousness of the two SARS-CoV-2 variants and the ratio between the transmissibility of the new SARS-CoV-2 variant and the previously prevalent SARS-CoV-2 variant. Figure 2 shows different epidemiological classes when ${𝓡}_{0_{1}}<1$ and ${𝓡}_{0_{2}}<1$. The SARS-CoV-2 variants disappear and the system reaches the disease free equilibrium point. Then in Figure 3 it can be seen that the solution reaches the endemic equilibrium point *EP* when ${𝓡}_{0_{1}}<1$ and ${𝓡}_{0_{2}}<1$. The preexistent SARS-CoV-2 variant vanishes. The last numerical simulation illustrates, in Figure 4, the case where ${𝓡}_{0_{2}}>{𝓡}_{0_{1}}>1$. The new highly transmissible SARS-CoV-2 variant still dominates over the preexistent variant, which disappears despite ${𝓡}_{0_{1}}>1$. The system reaches the endemic equilibrium point *EP*. When the new SARS-CoV-2 variant is introduced and this variant is more contagious, then the total number of infected, hospitalized, recovered, and deaths increases.

## 5. Conclusions

The proposed mathematical model assumes that individuals infected with one SARS-CoV-2 variant have full immunity against the other variant due to the adaptive immune response and that the immunity is lifelong. This is not well known and there are currently ongoing studies concerning this. If immunity is not lifelong then the model needs to be modified slightly. In this case, we would not expect the qualitative results to change, and the more transmissible SARS-CoV-2 variant would still take over the population and, in fact, at a much faster pace. The proposed mathematical model does not consider any vaccination program. Thus, in countries where vaccination is advancing quickly it is necessary to construct an alternative model that includes the vaccinated class. Nevertheless, even if vaccination programs are included we expect that similar qualitative conclusions would be reached since the new more transmissible SARS-CoV-2 variant will become the prevalent one based on the results presented here. Eventually, at some point, if all the population is vaccinated and the immunity is lifelong then both SARS-CoV-2 variants will vanish. This situation seems unlikely due to different factors such as, for example, the reluctance of a great number of people to get vaccinated.

We constructed a mathematical model based on a set of deterministic ordinary differential equations that describe the dynamics of the spread of two variants of SARS-CoV-2. The motivation for doing this is the current COVID-19 pandemic and, particularly, the recent detection of new SARS-CoV-2 variants that are more transmissible than the preexistent ones. The new SARS-CoV-2 variants have been of health public concern due to their higher infectiousness. The proposed model differs from previous models since it considers two different SARS-CoV-2 variants as well as asymptomatic cases for both variants. The analysis of the proposed mathematical model was conducted taking into account the higher transmission rate of a new SARS-CoV-2 strain and the subpopulation of asymptomatic carriers. We used the next generation matrix method to obtain two basic reproduction numbers ${𝓡}_{0}$. We proposed several theorems that established the necessary and sufficient conditions for the asymptotic global stability of the disease free and endemic steady states. We constructed some suitable Lyapunov functionals and used the LaSalle invariant principle to prove the global stability. The theoretical results show that there is competition between the SARS-CoV-2 variants, but the variant that persists is the one with the larger basic reproduction number ${𝓡}_{0_{2}}>{𝓡}_{0_{1}}$, which is the new SARS-CoV-2 variant. We performed numerical simulations that corroborated our analytical results. As we mentioned previously, these results help to support decisions in terms of health policies and to raise awareness about the risks of the introduction of new SARS-CoV-2 variants with higher transmission rates such the VOC-202012/01 of lineage B.1.1.7 or the 501Y.V2.

## Figures and Tables

**Table 1.** Mean values of parameters for the numerical simulations.

Parameter	Symbol	Value
Incubation period	*α* ^−1^	5.2 days [68,69]
Infectious period	*γ* ^−1^	7 days [68]
Hospitalization rate	*h* ^−1^	3.5 days × 0.04 [50,68,70]
Hospitalization period	*ρ* ^−1^	10.4 days [50,68,70]
Death rate (hospitalized)	*δ* ^−1^	10.4 days × 0.103 [65,71]
Probability of being asymptomatic	*a*	0.5 [1,72]
Recruiting rate	Λ	767.1 days^−1^ [73]
Death rate	*d*	0.00002378 days^−1^ [73]
